# Recent advances in adoptive cell therapy for cancer immunotherapy

**DOI:** 10.3389/fimmu.2025.1665488

**Published:** 2025-09-22

**Authors:** Jiameng Qian, Yuhua Liu

**Affiliations:** ^1^ Affiliated Hospital of Hangzhou Normal University, Hangzhou, Zhejiang, China; ^2^ Department of Clinical Laboratory, The Affiliated Hospital of Hangzhou Normal University, Hangzhou, Zhejiang, China

**Keywords:** next-generation ACT, CAR-NK, CAR-M, macrophage engineering, iPSC-derived cells

## Abstract

Adoptive cell therapy (ACT), a key direction in tumor immunotherapy, has achieved remarkable progress in recent years. This paper systematically reviews the current status and future trends of ACT, covering lymphokine-activated killer cells (LAK), tumor-infiltrating lymphocytes (TIL), cytokine-induced killer cells (CIK), dendritic cells (DC), T cell receptor-modified T cells (TCR-T), chimeric antigen receptor T cells (CAR-T), natural killer (NK) cells, chimeric antigen receptor-modified NK cells (CAR-NK), and the emerging CAR-M. The paper focuses on emerging technological approaches, including universal CAR structural optimization, iPSC-derived cell products, multifunctional CAR design, and AI-assisted antigen screening. It also compares differences among various cell therapies in antigen specificity, efficacy persistence, safety, and clinical application challenges. The core contribution of this paper lies in synthesizing recent research advances to propose strategies for addressing tumor heterogeneity, antigen escape, cell persistence, and therapeutic safety in ACT. This provides a reference for future personalized and precision cell therapy approaches.

## 1 An overview of adoptive immunotherapy

Adoptive Cell Transfer Therapy (ACT) involves isolating immune-active cells from a cancer patient, expanding and functionally characterizing them *in vitro*, and then reinfusing them into the patient. This aims to directly kill tumor cells or stimulate the body’s immune response to eliminate them.

Compared to traditional therapies, adoptive cell immunotherapy offers numerous advantages. It not only directly kills tumor cells but also mobilizes the body’s own immune function to suppress tumor growth, maintaining a relative dynamic equilibrium within the tumor’s unique microenvironment and halting its progression (1). Additionally, this therapy demonstrates high efficacy, low toxicity, strong targeting capabilities, and the benefit of memory-mediated immunity.

Early attempts to treat transplanted tumors in mice using T cells were primarily limited by the inability to effectively expand and utilize T cells *in vitro*. Consequently, early ACT employed allogeneic lymphocytes from highly immunized rodents for transplantation, demonstrating a degree of growth inhibition in established small tumors (2). Subsequent preclinical studies revealed a critical discovery highlighting the importance of host suppression factors: lymphocyte depletion via chemotherapy or radiation prior to cell infusion significantly enhanced the therapeutic efficacy of infused lymphocytes against established tumors (3). This pivotal finding charted a new course for subsequent tumor immunotherapy research.

The discovery of T-cell growth factor (interleukin-2) in 1976 provided a method for *in vitro* expansion of T lymphocytes without typically causing loss of effector function (4), overcoming the initial technical challenges of adoptive immunotherapy. To broaden the application of ACT for treating various human cancers, researchers began exploring strategies to genetically engineer lymphocytes to express anti-tumor receptors. Building upon mouse model studies (5), a 2006 study first demonstrated in humans that peripheral blood lymphocytes, through retroviral transduction, could express T cell receptors (TCRs) capable of recognizing the MART-1 melanoma-associated antigen, thereby mediating tumor regression (6). Subsequently, a breakthrough study in 2010 demonstrated that lymphocytes genetically modified to express chimeric antigen receptors (CARs) targeting the CD19 antigen on B-cell surfaces could achieve clinical remission in advanced B-cell lymphoma (7). These findings—whether harnessing naturally occurring antitumor T cells or genetically engineered antitumor T cells—laid the foundation for the further advancement of ACT in human cancer therapy.

In current clinical trials, ACT encompasses multiple types of cell therapies, primarily including: Lymphokine-Activated Killer Cells (LAK cells), tumor-infiltrating lymphocytes (TIL), cytokine-induced killer cells (CIK), dendritic cells (DCs), T cell receptor-engineered T cells (TCR-T cells), chimeric antigen receptor T cells (CAR-T cells), Natural Killer Cells (NK cells), Chimeric Antigen Receptor-Modified Natural Killer Cells (CAR-NK cells), and Chimeric Antigen Receptor Macrophages (CAR-M cells). Each of these cellular products possesses distinct immunological characteristics and mechanisms of action, demonstrating potential in treating various malignant tumors. With ongoing optimization of therapeutic strategies and deeper exploration of combination applications, ACT holds promise for further expanding the clinical prospects of tumor immunotherapy.

## 2 Adoptive immunotherapy types

### 2.1 LAK cell therapy

Lymphokine-activated killer cells (LAK cells) represent a significant breakthrough in the field of adoptive immunotherapy, first developed by Steven Rosenberg’s team at the National Cancer Institute in 1982 (8). The core process involves isolating peripheral blood leukocytes (PBL) from the patient’s blood, then activating and expanding them *in vitro* with high-dose recombinant interleukin-2 (IL-2) (9, 10). IL-2-stimulated LAK cells exhibit unique biological properties: they recognize multiple tumor cells through non-MHC-restricted mechanisms, exerting broad-spectrum antitumor effects without relying on antigen presentation (9). This characteristic made them a vital tool in early tumor immunotherapy.

The antitumor mechanism of LAK cells primarily relies on the perforin and granzyme system they secrete. When LAK cells come into contact with tumor cells, perforin forms transmembrane pores in the target cell membrane, facilitating the entry of granzyme B into the cell and activating the caspase cascade reaction, ultimately inducing programmed cell death in tumor cells (11). However, this killing mechanism lacks tumor-specific recognition, posing a potential risk of damage to normal tissues. This limitation has led to its declining use in contemporary tumor immunotherapy.

IL-2 plays a dual role in LAK cell therapy: on one hand, it promotes the *in vitro* expansion and activation of LAK cells by binding to the high-affinity IL-2 receptor (CD25) on effector cell surfaces; on the other hand, IL-2 also enhances the cytotoxic activity and cytokine secretion capacity of LAK cells. However, clinical practice indicates that the high doses of IL-2 required for treatment (typically exceeding 6×10^5 IU/kg) induce severe systemic toxicities, including hypotension, capillary leak syndrome, and multiple organ dysfunction (11). These dose-limiting toxicities significantly constrain the clinical applicability of this therapy.

In early clinical studies, LAK cell therapy demonstrated certain antitumor activity in patients with metastatic melanoma and renal cell carcinoma (11). However, this therapy has two critical limitations: First, the high doses of IL-2 required for treatment induce severe dose-limiting toxicities such as capillary leak syndrome. Second, LAK cells lack tumor-specific recognition capabilities, resulting in limited clinical efficacy and poor safety profiles. These limitations prompted researchers in the late 1980s to shift focus toward more targeted immunotherapy approaches. This shift in research direction holds significant scientific importance: the shortcomings of LAK therapy directly propelled a strategic transition in tumor immunotherapy from “nonspecific killing” to “specific recognition.” Researchers began attempting to isolate naturally occurring tumor-reactive lymphocytes from tumor tissues. This innovative approach laid a crucial foundation for the subsequent development of TIL cell therapy.

### 2.2 TIL cell therapy

TTILs are a type of immune cell isolated from the tumor microenvironment, representing a heterogeneous population rich in tumor-specific cytotoxic T lymphocytes and NK cells.

In 1988, Rosenberg first systematically reported TIL therapy in *Science* (12). The core technical approach of this therapy involves: surgically obtaining tumor tissue samples from patients, isolating TILs from these samples, selectively expanding them *in vitro* through IL-2 stimulation, and finally reinfusing large quantities of activated TILs back into the patient’s body (13). Compared to LAK cells, TILs demonstrate significantly enhanced tumor antigen recognition and targeted killing efficiency, primarily attributed to their TCRs having undergone prior selection and activation by tumor antigens (12).

In recent years, with the continuous advancement of tumor immunology research, TIL therapy has achieved significant breakthroughs in clinical translation. Multiple clinical studies have confirmed that TIL therapy demonstrates remarkable antitumor activity in patients with advanced melanoma (14, 15). For instance, a meta-analysis incorporating 13 clinical trials demonstrated that the combination of TIL ACT with IL-2 achieved an objective response rate (ORR) of 44% in melanoma patients, with some patients even achieving long-term progression-free survival (15). Notably, lifileucel (Amtagvi)—the world’s first TIL therapy granted accelerated approval by the FDA in February 2024—demonstrated a 65.2% objective response rate in PD-1-resistant advanced melanoma patients during pivotal trials, with a complete response (CR) rate of 31.8% (16, 17). Beyond melanoma, TIL therapy has demonstrated remarkable clinical potential in HPV-associated cervical cancer. A 2019 study revealed that isolating and expanding HPV-specific TILs from patient tumor tissues, followed by reinfusion, induced complete tumor regression in some patients and significantly prolonged survival (18).

Despite demonstrating breakthrough efficacy in solid tumor treatment, TIL therapy faces multiple challenges in clinical application: First, the therapy requires surgical acquisition of tumor tissue, limiting its applicability for patients inoperable for biopsy; Second, the traditional TIL preparation cycle spans 4–6 weeks, potentially delaying treatment for patients with rapidly progressing disease. Additionally, immunosuppressive characteristics of the tumor microenvironment—such as regulatory T cell infiltration and high PD-L1 expression—may compromise the persistence and antitumor activity of reinfused TILs (19). In the future, it may be necessary to combine TIL therapy with genetic modifications (such as PD-1 knockout), cytokine engineering, or combination therapies (such as immune checkpoint inhibitors, oncolytic viruses, etc.) to enhance its persistence and antitumor effects *in vivo*.

### 2.3 CIK cell therapy

In 1991, Schmidt-Wolf and colleagues first reported a novel type of immune effector cell—CIK cells (20). This cell population was generated by stimulating peripheral blood mononuclear cells (PBMCs) *in vitro* using anti-CD3 monoclonal antibodies combined with cytokines such as IL-2. Studies indicate that CIK cells possess unique biological characteristics: they exhibit both the antigen-specific recognition capability of T cells and the MHC-independent killing function of NK cells. This dual nature holds significant promise for their application in the field of tumor immunotherapy.

Studies indicate that adding IL-2 during short-term *in vitro* culture of human peripheral blood lymphocytes (PBLs) significantly promotes the proliferation and differentiation of effector NK cells and non-specific T cells, endowing them with LAK cell activity (21, 22). These IL-2-activated LAK cells exhibit unique antitumor properties: on one hand, they can effectively lyse fresh tumor cells *in vitro* through non-MHC-restricted mechanisms (23); on the other hand, *in vivo*, these cells demonstrate selective killing of tumor tissues while maintaining relative safety toward normal tissues. Furthermore, *in vitro*, CIK cells demonstrate enhanced proliferative potential. Experimental data indicate that under optimized culture conditions, CIK cells can expand over 1000-fold (20, 24). This characteristic provides an ample cellular source for clinical applications, representing a significant advantage for adoptive immunotherapy.

CIK cell therapy has emerged as a significant approach in tumor immunotherapy, garnering widespread attention due to its low toxicity and favorable safety profile. Extensive clinical studies demonstrate that this therapy has achieved remarkable progress in treating both hematologic malignancies and solid tumors (25) (26–28). In hematologic malignancies, CIK cells demonstrate potent cytotoxic activity against multiple myeloma and leukemia cells. When combined with stem cell transplantation, this approach effectively reduces disease recurrence rates (25). Clinical studies on non-Hodgkin lymphoma (NHL) have also confirmed the favorable tolerability and therapeutic efficacy of CIK cell therapy (26, 27).

In the field of solid tumor therapy, CIK cell therapy demonstrates broader application value. Advanced gastric cancer patients undergoing CIK cell therapy experienced significant improvements in immune function, reduced tumor burden, and enhanced quality of life. Combining this therapy with chemotherapy produced synergistic effects, prolonging patient survival (29, 30). For hepatocellular carcinoma (HCC) patients unresponsive to conventional treatments, minimally invasive procedures combined with CIK cell immunotherapy significantly extended recurrence-free survival (31, 32). Adjuvant CIK cell therapy after breast cancer surgery markedly improves patient prognosis and extends overall survival (33). Furthermore, in nasopharyngeal carcinoma treatment, the median overall survival in the chemotherapy plus CIK cell therapy group (32 months) significantly outperformed the chemotherapy-only group (9 months) (34).

However, CIK cell therapy still faces several pressing challenges. Its non-specific killing mechanism results in insufficient targeting, and issues such as high cellular heterogeneity after *in vitro* expansion constrain the standardized implementation and clinical promotion of this therapy. Future research should focus on optimizing culture systems to enhance cellular homogeneity and exploring combination strategies with other immunotherapy approaches to further improve the clinical efficacy of CIK cell therapy (28). Currently, DC-CIK combination therapy shows promising prospects, where dendritic cells (DCs) effectively present tumor antigens, significantly enhancing the tumor-specific killing capacity of CIK cells (35).

### 2.4 DC therapy

DCs are the most potent antigen-presenting cells in the body, playing a pivotal role in initiating and regulating both innate and adaptive immune responses. DC cell therapy primarily leverages their potent antigen uptake, processing, and presentation capabilities. By isolating DC precursor cells from a patient’s peripheral blood and differentiating them into mature DCs *in vitro*, while simultaneously exposing them to tumor-marked tissue or synthetic antigen peptides, these DCs can convey tumor markers to T lymphocytes. Upon reinfusion, this process initiates the body’s specific immune response against tumors (36). Following antigen uptake and processing, mature dendritic cells activate naive T cells (Th0) and further induce their proliferation and differentiation into effector T cells, including CD4+ helper T cells and CD8+ cytotoxic T cells, thereby initiating the body’s adaptive immune response (37–39). It can be said that dendritic cells play a central role in initiating, regulating, and sustaining immune responses.

Additionally, DCs direct the immune system to attack tumor cells. After recognizing and internalizing tumor-associated antigens via their surface pattern recognition receptors (PRRs), DCs process these antigens to form MHC-antigen peptide complexes expressed on the cell surface (36). Mature DCs then migrate to secondary lymphoid organs, where they activate T cell immune responses through antigen presentation and co-stimulatory signals (37). Specifically, the MHC class I-antigen peptide complexes on DC surfaces bind to the TCR of CD8+ T cells. Under the influence of co-stimulatory molecules (CD80/CD86) and cytokines (e.g., IL-12) (40), this interaction induces CD8+ T cells to differentiate into cytotoxic T lymphocytes (CTLs) with tumor-killing activity. These CTLs eliminate tumor cells through the following mechanisms: releasing perforin and granzyme to form pores in the tumor cell membrane and induce apoptosis; triggering death receptor-mediated apoptosis via the Fas/FasL pathway; and secreting cytokines such as IFN-γ to suppress tumor growth (36, 41). Concurrently, DC-activated CD4+ helper T cells further amplify the immune response by secreting cytokines (e.g., IL-2, IFN-γ) and help maintain the function of CTLs and memory T cells (36, 42). This DC-initiated, multi-layered immune response constitutes the core mechanism of the body’s antitumor immune defense.

In addition, after inducing DC maturation *in vitro*, they are mixed with the patient’s own lymphocytes to enhance the stimulation effect, prompting the patient’s T cells to generate more cytotoxic T lymphocytes. These enhanced cytotoxic T lymphocytes are then reinfused into the patient to directly kill tumor cells. However, overall, DC cell therapy remains in a phase of ongoing exploration and refinement.

With the continuous advancement of cellular medicine, DC-based immunotherapy has achieved rapid progress within just a few years. The first clinical trial of a DC vaccine commenced in 1996, utilizing tumor antigen-loaded peptides to activate patients’ T cells for postoperative immune reconstruction in prostate cancer (43). This pioneering study laid the foundation for subsequent clinical applications of DC vaccines. In 2010, the autologous prostate cancer vaccine Sipuleucel-T (Provenge), based on DC technology, received FDA approval for commercialization, becoming the world’s first therapeutic tumor vaccine utilizing dendritic cells. This therapy successfully activated specific anti-tumor T-cell responses by ex vivo conjugating patient dendritic cells with prostate acid phosphatase (PAP) antigen. Key clinical trial data demonstrated that Sipuleucel-T significantly extended overall survival in patients with metastatic castration-resistant prostate cancer, with a median survival increase of 4.1 months. This breakthrough achievement marked the dawn of a new era in cancer immunotherapy (44).

In recent years, the development of DC vaccines has shown rapid growth. Statistical data indicates that 34 DC vaccine studies were published between 2017 and 2019, with 59 studies ongoing during the same period. From 2015 to 2020, a total of 42 DC vaccine products were launched globally (45). These advancements demonstrate that DC vaccines have emerged as a highly promising personalized cancer treatment platform, with their combination with existing immunotherapies expected to further enhance clinical efficacy. Notably, DC vaccines demonstrate exceptional efficacy in melanoma treatment. Results from a Phase III clinical trial (NCT02993315) published in 2024 revealed that autologous DC vaccines achieved a 2-year overall survival rate of 84.7% in patients with stage III B/C melanoma, significantly outperforming the control group (46). Furthermore, recent studies have demonstrated that adenovirus-mediated delivery of transcription factors PU.1, IRF8, and BATF3 (PIB) can directly reprogram tumor cells *in vivo* into antigen-presenting DC-like cells (47). This *in situ* reprogramming technique not only overcomes the limitations of traditional DC therapies—specifically cell collection and *in vitro* expansion—but also significantly enhances antitumor immunity.

However, DC immunotherapy still faces numerous challenges, such as limited migration efficiency of DCs within the tumor microenvironment and impaired antigen presentation efficiency due to immunosuppressive microenvironments. The latest research by Lin Yuan and Liang Jiankai’s team at Sun Yat-sen University revealed that the oncolytic virus M1 (OVM1) can significantly enhance the antitumor efficacy of DC vaccines by downregulating SIRPα on DC surfaces and CD47 on tumor cell surfaces, thereby relieving their immunosuppressive effects on DC vaccines (48). This discovery provides new insights for optimizing DC vaccines. In the future, strategies such as *in situ* reprogramming technology, combined immunotherapy, and genetically engineered DCs may significantly enhance efficacy and accessibility. However, their safety, long-term effects, and clinical feasibility still require systematic validation. Therefore, while DC immunotherapy holds great promise, it necessitates further optimization in standardization, efficiency, and efficacy enhancement.

### 2.5 TCR-T cell therapy

Since the 21st century, breakthroughs in genetic engineering technology have propelled the development of TCR-T therapy. TCR-T therapy employs genetic modification to engineer patients’ T cells to express TCRs that specifically recognize tumor antigens, enabling precise targeting and elimination of cancer cells (49). Compared to non-specific cell therapies like LAK and CIK, TCR-T possesses precise antigen recognition capabilities, allowing it to specifically target tumor-associated antigens (49). The core of this technology lies in the TCR molecule, which acts as the “gatekeeper” of T cell function. Its interaction mechanism with the MHC determines the intensity and quality of the antitumor immune response (50).

TCR-T cell therapy demonstrates unique biological advantages and clinical potential in the treatment of solid tumors. From a molecular perspective, the TCR molecule—a key member of the immunoglobulin superfamily—forms a heterodimer composed of α and β chains. This heterodimer precisely couples with the CD3 complex (comprising four signaling chains: γ, δ, ϵ, and ζ) to create the complete TCR-CD3 complex. This structural feature endows TCR-T cells with highly specific recognition of tumor antigens (50). Regarding mechanism of action, TCR-T cells retain the innate T cell activation pathway. Through specific binding of TCR to MHC-antigen peptide complexes (particularly recognition of MHC class II molecules), they not only directly activate the cytotoxic function of CD8+ T cells but also establish a more comprehensive and durable antitumor immune response through the synergistic action of CD4+ helper T cells (51, 52). Compared to CAR-T cells, which primarily recognize surface-specific antigens on tumors (53), the greatest advantage of TCR-T cells lies in their ability to recognize intracellular tumor antigens presented by MHC molecules. This includes tumor-specific mutated antigens (such as neoantigens) and virus-associated antigens (e.g., HPV E6/E7 proteins), significantly broadening the spectrum of targetable antigens (54). This unique antigen recognition characteristic enables TCR-T cells to demonstrate superior tissue infiltration and persistence within the solid tumor microenvironment.

In recent years, TCR-T cell therapy has achieved significant progress in clinical translation and commercialization. The landmark research conducted by the Rosenberg team first demonstrated the therapeutic potential of TCR-T cells in melanoma patients (6). TCR-T therapy targeting the NY-ESO-1 antigen showed substantial objective response rates in patients with synovial sarcoma and melanoma, with some achieving complete remission (55). Furthermore, a TCR-T cell therapy targeting the HPV-16 E7 protein (NCT02858310) demonstrated objective responses in 6 out of 12 patients in a Phase 1 clinical trial for metastatic HPV-associated epithelial carcinoma, confirming the clinical feasibility of viral antigen-targeting strategies (56). In 2022, Kimmtrak (tebentafusp), the world’s first TCR-T therapy, received FDA approval for treating HLA-A*02:01-positive metastatic uveal melanoma patients. Phase III clinical trial data showed a median overall survival (OS) of 21.7 months, significantly longer than the 16.0 months observed in the control group (57). In August 2024, Adaptimmune’s TCR-T product Tecelra (afami-cel) received FDA accelerated approval, becoming the first TCR-T therapy for synovial sarcoma. Its SPEARHEAD-1 trial demonstrated an objective response rate (ORR) of 43%, a complete response rate of 4.5%, and sustained responses exceeding 12 months in 39% of responders (58). In China, Xiangxue Pharmaceutical’s TAEST16001 injection has been designated as a breakthrough therapy. Its Phase I clinical trial for advanced soft tissue sarcoma demonstrated an ORR of 41.7%, and the company is currently advancing Phase II studies (59).

However, TCR-T therapies still face challenges such as HLA restriction and off-target toxicity. For example, a TCR-T therapy targeting MAGE-A3 caused severe neurotoxicity due to cross-reactivity (60). Future development of TCR-T therapies should focus on discovering novel targets (such as personalized neoantigens), combining with immune checkpoint inhibitors (such as PD-1/PD-L1 blockers), and developing universal TCR-T products (60).

### 2.6 CAR-T cell therapy

TCR-T therapy, constrained by HLA restriction and potential off-target toxicity, has spurred the development of more flexible targeting strategies. Among these, CAR-T cells have rapidly emerged as a research hotspot due to their unique advantage of bypassing MHC dependence and directly recognizing tumor surface antigens. The CAR structure has undergone multiple generations of iterative optimization: the extracellular domain employs a single-chain variable fragment (scFv) for antigen-specific recognition, the transmembrane domain typically originates from CD8 or CD28 (61), and the intracellular signaling domain incorporates CD3ζ along with one or more co-stimulatory molecules (e.g., CD28, 4-1BB), collectively forming a complete T-cell activation pathway (62). Currently, second-generation CAR-T cells dominate clinical applications, demonstrating significant efficacy in hematologic malignancies (62). With advancing research, multifunctional CAR design has gained prominence. By fusing multiple co-stimulatory factors (CD28, 4-1BB, OX40) within the intracellular signaling domain, or adding cytokine expression modules (IL-12, IL-15), and incorporating logic gates (AND, OR, NOT) to enable multi-antigen recognition, these designs significantly enhance the persistence and precision of antitumor responses (63). For instance, “armored CAR-T” enhances local immune effects through autocrine cytokine secretion, while “bispecific CAR” mitigates the risk of immune escape triggered by single-target mutations (64).

In recent years, CAR-T cell therapy has made significant advances in the treatment of B-cell malignancies. Multiple clinical trials have demonstrated its efficacy in refractory/relapsed B-cell acute lymphoblastic leukemia (B-ALL) and NHL. refractory B-ALL and NHL. These studies primarily target CD19 (65, 66), CD20 (67), or CD30 (68). Among these, CD19-targeted CAR-T cells achieved CR rates of 70%-94% in B-ALL patients (65, 69). In 2017, the world’s first CAR-T product received regulatory approval for market launch. Subsequently, China also approved Axicabtagene Ciloleucel and Rixotumab Tigolizumab for the treatment of large B-cell lymphoma (70–72). Furthermore, CAR-T therapy has demonstrated preliminary efficacy in other B-cell malignancies such as mantle cell lymphoma and marginal zone lymphoma (7, 73, 74).

However, the application of CAR-T therapy in solid tumors still faces significant challenges, including tumor heterogeneity, immunosuppressive microenvironments, and insufficient CAR-T cell infiltration (74–76). Despite these hurdles, researchers are actively exploring novel targets such as CLDN18, CD276, and KRAS. Research on CLDN18 targeting has surged by 400% since 2021, demonstrating CAR-T’s potential for solid tumors (77–79). Solid tumors are also emerging as another frontier for CAR-T cell therapies, particularly demonstrating novel therapeutic potential in melanoma. A research team at the University of California, Los Angeles developed a novel CAR-T cell therapy targeting the TYRP1 protein, which is highly expressed in approximately 30% of cutaneous melanomas and up to 90% of rare subtypes such as uveal melanoma (80). Preclinical studies demonstrate that these CAR-T cells effectively eradicate cancer cells without causing severe side effects, and clinical trials are currently planned (80).

Therefore, future CAR-T therapy research should focus on exploring new targets with high expression levels and low expression in normal tissues, optimizing CAR structural design, and integrating microenvironment modification strategies. Overall, while its potential is immense, overcoming microenvironment barriers and safety challenges remains essential.

### 2.7 NK cell therapy

NK cells, as key effector cells of the innate immune system, play an irreplaceable role in tumor immune surveillance and antitumor immune responses. Unlike T cells, NK cells possess unique MHC-independent killing properties, enabling them to recognize and eliminate tumor cells without prior antigen sensitization. This characteristic makes them a significant focus of research in the field of tumor immunotherapy (81). In recent years, advances in genetic engineering technologies have demonstrated the broad potential of engineered NK cells (such as CAR-NK) in targeted tumor therapy.

The antitumor mechanisms of NK cells exhibit multidimensional characteristics. First, through antibody-dependent cell-mediated cytotoxicity (ADCC), the FcγRIII (CD16) on the surface of NK cells can bind to tumor-specific antibodies, enabling specific killing of tumor cells. This mechanism has become a crucial foundation for various antibody drug therapies (81). Second, NK cells directly kill target cells by releasing effector molecules such as perforin and granzyme. Notably, this killing exhibits high selectivity, sparing the NK cells themselves from damage—a property that ensures safety for clinical applications (82). Third, TRAIL and Fas ligand expressed on NK cell surfaces can induce apoptosis in tumor cells expressing their respective receptors (83). This death receptor pathway, together with the perforin pathway, constitutes the NK cell killing network. Concurrently, NK cells can traverse the blood-brain barrier to infiltrate brain tumor tissues (84). Furthermore, NK cells can interact with other immune cells (such as DCs, T cells, and macrophages) by secreting various chemokines, growth factors, and cytokines, thereby activating the adaptive immune response and inhibiting tumor progression (82).

NK cell therapy can be further categorized into autologous NK cell therapy, allogeneic NK cell therapy, CAR-NK cell therapy, and monoclonal antibody-based NK cell therapy. Autologous NK cell therapy utilizes the patient’s own NK cells, thereby avoiding immune rejection risks. However, NK cells from cancer patients are often functionally suppressed, which compromises therapeutic efficacy (85). Allogeneic NK cell therapy employs NK cells from healthy donors, overcoming functional deficiencies while enhancing antitumor activity through KIR-ligand mismatch mechanisms and exhibiting lower GVHD risk (85). The most groundbreaking approach is CAR-NK cell therapy (86), which combines CAR engineering with NK cell characteristics to demonstrate unique advantages: First, its safety profile significantly outperforms CAR-T therapy. NK cells do not secrete key inflammatory mediators like IL-6, drastically reducing CRS risk. Additionally, they are not HLA-restricted, resulting in extremely low GVHD incidence (87). Second, NK cells possess multiple recognition mechanisms. Beyond CAR structures, they can identify tumor cells through innate receptors such as DNAM-1 and NKG2D, enhancing targeting precision (88, 89). Finally, NK cells are widely accessible, obtainable from peripheral blood, umbilical cord blood, or NK cell lines, facilitating large-scale production (88, 89). Recent studies have also extended CAR-NK cell survival *in vivo* through genetic modification, enhancing their infiltration capacity into solid tumors, and engineered enhanced CAR-NK cells resistant to immunosuppressive microenvironments (90). Furthermore, NK and T cell activity is regulated by multiple surface inhibitory receptors, including NKG2A, LAG3, TIM-3, and TIGIT (91). Currently, several monoclonal antibodies are undergoing clinical evaluation to block these immune checkpoints. Research data indicates that blocking these receptors may unleash NK cells, potentially yielding positive therapeutic effects (91).

In recent years, NK cell therapy has achieved breakthrough progress in the field of tumor immunotherapy, with its application expanding from hematologic malignancies to solid tumors and infectious diseases. In hematologic malignancies, allogeneic NK cell infusion has demonstrated durable antitumor responses in patients with acute myeloid leukemia (AML) (92), while CAR-NK cell therapy has shown favorable safety profiles and preliminary efficacy in clinical studies for multiple myeloma (MM) (93). For hepatocellular carcinoma treatment, research from Chonnam National University in South Korea confirmed that locally administered high-dose autologous NK cells combined with hepatic arterial infusion chemotherapy (HAIC) significantly extended progression-free survival (PFS) and overall survival (OS) in patients, achieving a disease control rate as high as 80% (94). Regarding infectious diseases, studies indicate NK cell therapy may positively impact chronic hepatitis B and HIV infection (95, 96). Research on SARS-CoV-2 infection also suggests NK cells hold potential as a key component of COVID-19 immunotherapy (97). Notably, In 2025, research published by Academician Cao Xuetao’s team in Signal Transduction and Targeted Therapy revealed that Neo-2/15-modified CAR-NK cells significantly enhance antitumor activity in solid tumors such as pancreatic and ovarian cancers by activating the c-Myc/NRF1 signaling pathway (98), offering a novel strategy to overcome immune suppression in the tumor microenvironment.

CAR-NK cell therapy, as a significant advancement in NK cell treatment, has achieved remarkable clinical outcomes in recent years. Currently, multiple CAR-NK products worldwide have entered clinical stages, such as Fate Therapeutics’ CD19 CAR-iNK cell therapy FT522 and China’s Zhongsheng Suyuan’s NCR-300 (99, 100). Regarding solid tumor treatment, a 2025 study revealed that knocking out the SMAD4 gene significantly enhances CAR-NK cells’ resistance to TGF-β inhibition, improving their infiltration and killing capacity within the tumor microenvironment (101). Additionally, off-the-shelf CAR-NK cells derived from induced pluripotent stem cells (iPSCs) are gaining prominence due to their scalability and ease of genetic modification, offering potential solutions to the high costs associated with personalized therapies (100).

Despite the promising prospects of CAR-NK therapy, several challenges remain. First, NK cells exhibit limited *in vivo* persistence, typically surviving only 1–4 weeks, which constrains their long-term efficacy. Second, immunosuppressive factors in the solid tumor microenvironment (such as TGF-β and adenosine) impair CAR-NK cell function (102). Lastly, CAR-NK transduction efficiency remains low, and large-scale production still faces technical bottlenecks (103). Future research should focus on optimizing CAR structural design (e.g., multi-target CARs), enhancing cellular metabolic adaptability (e.g., IL-15 co-expression), and developing universal CAR-NK products to further improve their clinical applicability. With breakthroughs in these technologies, CAR-NK therapy is poised to become a significant breakthrough in tumor immunotherapy following CAR-T.

### 2.8 CAR-M cell therapy

Due to the complex tumor microenvironment, physical barriers, and immunosuppressive factors in solid tumors, CAR-T and CAR-NK cell therapies face numerous challenges in treating solid tumors (102). Consequently, researchers have begun focusing on other types of immune cells and attempting to engineer them using CAR technology to overcome these limitations. Macrophages, with their potent tumor infiltration capabilities, superior phagocytic functions, and antigen-presenting properties, have emerged as a promising candidate immune cell type (104). As progress in this field continues, CAR-M cells have been developed, offering new hope for the immunotherapy of solid tumors.

The basic structure of CAR-M is similar to CAR-T, comprising an antigen recognition domain (typically scFv), a transmembrane domain, and an intracellular signaling domain. However, CAR-M often incorporates signaling elements such as FcRγ, CD3ζ, CD28, or CD40 to activate phagocytosis and inflammatory cytokine release signaling pathways. For instance, the FcRγ and DAP12 adaptor proteins activate phagocytosis via Syk kinase; the PI3K/AKT and NF-κB pathways drive the release of inflammatory mediators (TNF-α, IL-12, etc.), thereby enhancing the remodeling of the tumor microenvironment (105–107). Furthermore, the CAR-M-induced M1 polarization state helps sustain the antitumor immune cycle (108, 109). Through these mechanisms, CAR-M cells play a crucial role within the tumor microenvironment, offering a novel immunotherapeutic approach for treating solid tumors.

CAR-M therapy is an emerging immunotherapy technology designed to enhance immune responses against solid tumors through engineered macrophages. This approach combines CAR technology with the innate properties of macrophages (104), demonstrating potential in overcoming challenges in solid tumor treatment. Initial first-generation CAR-M cells primarily employed gene transfection techniques to express tumor-specific CAR structures on the macrophage surface, thereby enhancing their ability to recognize and phagocytose tumor cells (110). However, first-generation CAR-M cells exhibited limitations such as low *in vitro* expansion efficiency, suboptimal transfection rates, and limited *in vivo* persistence. With ongoing technological advancements, second-generation CAR-M cells have been optimized in multiple aspects. First, improvements in vector systems—such as the use of adenovirus vectors or electroporation methods—significantly enhanced cell engineering efficiency. Concurrently, the introduction of co-stimulatory signals (e.g., CD28 or 4-1BB) further boosted CAR-M functionality, particularly in activating and amplifying immune responses. Second-generation CAR-M not only improved cellular efficiency but also overcame several technical bottlenecks faced by first-generation CAR-M (110, 111). Recently, the emergence of third-generation CAR-M cells has brought further innovations. In CAR design, researchers have incorporated cytokine expression modules, such as Granulocyte-Macrophage Colony-Stimulating Factor (GM-CSF) or IL-12, along with autocrine activation signals (110). These enhancements have improved CAR-M cell survival, proliferation, and immune activation within the tumor microenvironment, offering new avenues for their application in complex solid tumors (112).

Research on CAR-M began in 2020, when Klichinsky et al. first reported the efficacy of humanized CAR-M against HER2-positive tumors *in vitro* and in animal models (109). In 2021, Carisma Therapeutics initiated the first clinical trial of HER2-targeted CAR-M, marking its entry into the clinical validation phase (89). In 2024, a team from Peking Union Medical College Hospital developed c-MET-targeted CAR-M cells, demonstrating significant efficacy in pancreatic cancer models and advancing into preclinical studies (113). By 2025, Carisma announced Phase I clinical results showing disease stabilization in some patients with favorable safety profiles (114). Concurrently, strategies for generating CAR-M cells via induced pluripotent stem cell (iPSC) differentiation gained traction, offering potential for standardized and scalable production (115).

By comparing CAR-M cell therapy with CAR-T and CAR-NK therapies (**Table 1**), its unique advantages and limitations become more apparent. While CAR-T therapy has achieved breakthroughs in hematologic malignancies (116), its application in solid tumors remains constrained by difficulties in penetrating tissue barriers, the impact of immunosuppressive microenvironments, and the risk of cytokine storms (102). CAR-NK therapy exhibits lower immunotoxicity and natural killer activity but has limited persistence and expansion capacity *in vivo* (102, 117). In contrast, CAR-M cells can actively infiltrate tumor core regions and initiate T-cell responses through antigen presentation, with a lower incidence of CRS, making them theoretically more suitable for solid tumor immunotherapy (118, 119).

**Table 1:** Comparison of CAR-M, CAR-T, and CAR-NK.

Type	CAR-M	CAR-T	CAR-NK
Primary Effect	• Phagocytosis • Antigen Presentation • TME Remodeling, Amplifying Paratumor Effects	• Granzyme/Perforin Direct Cytotoxicity • Amplification	• Innate Cytotoxicity • IFN-γ, HLA Non-Restricted Portion
Barrier to solid tumors	Innate chemotaxis to tumor; resistant to hypoxia/acidity; can “deplete stroma”	Restricted migration/survival, requires armoring	Migrates well but exhibits limited persistence in solid tumors
*In-vivo* amplification	No amplification; repeated dosing or *in vivo* programming is required	Strong amplification and persistence (advantage in hematologic malignancies)	Generally, not long-lasting, often requiring repeated administration
Security Map	CRS/ICANS carries a lower risk, with localized inflammation being common	CRS/ICANS management is mature but remains a core risk	CRS risk is relatively low
Manufacturing and Accessibility	mRNA/LNP exhibits significant potential for *in vivo* programming	Complex and expensive	Can be made into a “universal” product, but consistency/persistence is a challenge.

However, CAR-M therapy still faces several challenges. Macrophages may be induced to adopt a pro-tumor M2 phenotype within the tumor microenvironment (120), thereby diminishing their anti-tumor function. Furthermore, the preparation and expansion techniques for CAR-M cells require further optimization to achieve standardized and scalable production. Utilizing iPSCs as a uniform, stable cell source enables large-scale *in vitro* expansion and differentiation into NK cells, macrophages, or even T cells, followed by CAR modification. This approach facilitates the development of “off-the-shelf” immune cell therapies. Existing research demonstrates that iPSC-derived CAR-NK and CAR-M cells exhibit robust antitumor activity *in vitro*, coupled with enhanced controllability and product consistency, laying the groundwork for future clinical translation (121).

In the future, CAR-M therapy holds promise for combination use with other immunotherapy modalities (such as CAR-T, immune checkpoint inhibitors, etc.) (122), leveraging synergistic effects to further enhance treatment efficacy. Research indicates that cytokines from CAR-T cells, such as IFN-γ and GM-CSF, can convert macrophages into the M1 phenotype, thereby amplifying the cytotoxicity of CAR-M cells (123). This mechanism offers a novel therapeutic strategy for combining CAR-M and CAR-T cells, potentially improving overall efficacy in solid tumor treatment. Furthermore, CAR-M technology is being explored for non-neoplastic disease applications. For instance, in 2025, a research team from Huazhong University of Science and Technology developed microneedle-delivered CAR-M (CAR-eM) for treating intervertebral disc degeneration. This marked the first extension of CAR-M therapy into non-tumor disease areas, significantly broadening its application prospects (124). This advancement not only demonstrates the potential of CAR-M therapy but also paves the way for its application in more clinical diseases.

### 2.9 ACT therapy comparison

Throughout the development of ACT, while the overall strategy relies on modifying or expanding immune cells in vitro before reinfusion to achieve antitumor effects, different cellular platforms exhibit significant differences in biological characteristics, recognition mechanisms, and clinical application prospects (**Table 2**).

**Table 2:** ACT therapy comparison.

Type	Cell source	Main target antigen	Indications	Advantages	Limitations
LAK Cell Therapy	Autologous peripheral blood lymphocytes	Non-specific	Early studies in solid tumors	Simple manufacturing	Limited efficacy; noticeable side effects
TIL Cell Therapy	Patient-derived tumor T cells	Tumor-specific antigens	Melanoma, some solid tumors	High specificity; can recognize multiple antigens	Long expansion time; limited indications; complex manufacturing
CIK Cell Therapy	Peripheral blood mononuclear cells expanded ex vivo	Non-specific, MHC-independent	Solid tumors, some hematologic malignancies	Rapid expansion; low toxicity	Lacks high specificity; limited efficacy
DC Cell Therapy	Patient-derived monocytes differentiated into DCs	Tumor-associated antigens or peptides	Solid tumors, some hematologic malignancies	Can prime and activate T-cell responses; personalized immunotherapy	Limited *in vivo* persistence; manufacturing complex; variable efficacy
TCR-T Cell Therapy	Autologous or allogeneic T cells	Tumor-specific peptide-MHC complexes	Solid tumors (melanoma, liver cancer, etc.), some hematologic malignancies	Can recognize intracellular antigens, expanding target range	HLA-restricted; potential off-target toxicity
CAR-T Cell Therapy	Autologous or allogeneic T cells	Tumor-associated antigens, e.g., CD19, BCMA	B-cell malignancies (ALL, DLBCL), multiple myeloma	High specificity and cytotoxicity; durable *in vivo* expansion	Cytokine release syndrome (CRS), neurotoxicity; limited efficacy in solid tumors; long manufacturing time
NK Cell Therapy	Autologous/allogeneic peripheral blood NK or iPSC-derived NK cells	NKG2D, CD19, HER2, etc.	Hematologic malignancies, solid tumors	Can be used allogeneically; low GVHD risk; low short-term toxicity	Short *in vivo* persistence; limited expansion
CAR-NK Cell Therapy	Short *in vivo* persistence; limited expansion	CD19, CD22, HER2, EGFR, etc.	Hematologic malignancies, some solid tumors	Allogeneic use; low GVHD and CRS risk	Less durable than CAR-T; limited *in vivo* expansion
CAR−M Cell Therapy	Monocyte/macrophage-derived	HER2, CD19, MUC1, etc.	Mainly solid tumors	Can phagocytose tumor cells and activate immune response; penetrate tumor microenvironment	Early-stage technology; limited persistence and expansion

## 3 Future prospects for ACT

Although ACT has demonstrated clinical benefits in hematologic malignancies and certain solid tumors, advancing from “feasible” to “widespread adoption” requires simultaneous technological evolution in manufacturing, product formulation, precision targeting, and safety control. First, automated cell manufacturing will become the watershed for cost and quality. Closed-system, single-use GMP automated production lines are achieving streamlined, robotic workflows for “expansion-purification-filling-freezing,” while online process analysis (PAT) and real-time release testing (RTRT) reduce turnaround times. Electronic batch records and model predictive control (MPC) enable early warning and correction before batch-to-batch variability or cellular phenotype drift occurs (125). Consequently, both the cost of goods sold (CoGS) and batch-to-batch variability can be reduced simultaneously, laying the foundation for scalable accessibility.

Second, universal (off-the-shelf) cell platforms are reshaping the supply model. Products like CAR-NK, γδT, and CAR-M, derived from donor sources or iPSC master libraries, achieve “immune stealth” and “low rejection” through gene editing (e.g., knocking out TRAC/B2M/CIITA or introducing HLA-E or CD47), significantly reducing waiting times and enhancing product consistency. Compared to autologous preparation, off-the-shelf platforms facilitate multicenter consistency validation and cost-sharing (126). However, their long-term chimeric persistence and *in vivo* longevity require reinforcement through cytokine armoring (e.g., IL-15), chemokine receptor engineering, and repeated dosing regimens.

Moreover, personalized ACT strategies will shift from an “antigen-centric” approach to a “patient ecosystem-centric” one. By integrating tumor multi-omics and spatial omics profiling, multidimensional characterization of “antigens-microenvironment-metabolism-immune history” can be completed prior to treatment, enabling selection of single/multi-target combinations and dosing sequences. The dual approach represents an effort to overcome tumor heterogeneity and antigen escape. For instance, Linfu and Yao et al. designed bispecific CAR-T cells that simultaneously targeting FAP and GPC3, showing the evidence of their ability to prevent antigen evasion and control heterogeneous HCC (127). In receptor engineering, affinity tuning and logic gates (AND/OR/NOT) will reduce off-target effects and antigen escape; on the cellular chassis, prioritizing enrichment of T_SCM/T_CM-like memory lineages or maintaining persistence through metabolic reprogramming (enhancing OXPHOS/mitochondrial mass) will be pursued; At the microenvironment level, introduce homing axes (e.g., CXCR2/CCR7) and tolerance modules (e.g., dominant negative TGFβR, PD-1:CD28 signal switching) to counter inhibitory signals. Sequential combination with immune checkpoint inhibitors, oncolytic viruses, and radiotherapy/chemotherapy will further amplify “antigen diffusion” and distant effects (128).

Synthetic biology-based CAR design provides a systemic solution for safety and controllability. Switchable adapter CARs (e.g., replaceable bridging molecules) enable the same cell to “swap ammunition” for different targets and rapidly remove ligands during adverse reactions to achieve “pharmaceutical-grade shutdown.” Intelligent circuits (synNotch, inhibitory iCAR, gated CAR) release killing only when specific microenvironment signals are satisfied. Armored CARs co-express IL-12/IL-18/IL-7/CCL19 to “self-amplify” local immunity, or achieve tumor-selective activation via protease-cleavable masks or hypoxia-inducible promoters. Parallel suicide switches (e.g., iCasp9) provide “one-click termination” safeguards for extreme scenarios (129). For the particularly challenging barrier issues in solid tumors, triggering efficiency under low-antigen conditions can be enhanced by optimizing transmembrane and endocytic signaling domains. This approach can be extended to CAR-M cells, making their “recognition-phagocytosis-presentation-microenvironment remodeling” cascade more efficient.

Finally, the application of artificial intelligence (AI)-assisted antigen screening is revolutionizing the development model of adoptive cell therapies. AI permeates the entire process from target identification to manufacturing. At the discovery stage, AI can screen for “tumor-specific, low-expressed in normal tissues” candidate antigens from transcriptomic/proteomic/glycomic and spatial omics data, while predicting off-target effects and organ toxicity (130). In molecular design, AI enables multi-objective optimization of scFv structural stability, signaling domain combinations, cytokine profiles, and immunodynamics (131, 132). In manufacturing, digital twins facilitate transfer learning from batch data to guide parameter settings and batch release thresholds, significantly reducing trial-and-error costs during development-to-production transitions (133). The integration of AI and automation will propel ACT from a “craft workshop” to a “data-driven industrial process”.

In summary, the next phase of ACT will not rely on breakthroughs in any single dimension, but rather on the synergistic evolution of “automated manufacturing, universal platforms, personalized strategies, synthetic biology CARs, and AI optimization.” The upstream segment significantly enhances treatment accessibility and preparation consistency through off-the-shelf cell products and closed production lines. The midstream leverages multimodal patient profiling and logic gate-driven CAR design to tailor treatment precision—specifying “which patient populations, at what timing, and with what intensity” receive therapy. The downstream employs strategies like intelligent synthetic circuits, switchable adapters, and safety switches to build a “controllable, reversible, and scalable” cell therapy system. Regulatory advancements, driven by the deepening application of Quality by Design (QbD) principles and the maturation of Real-World Evidence (RWE), position ACT to expand beyond oncology into select non-oncological indications while maintaining safety control. This progression paves the way for realizing the vision of cell therapy that is both accessible and precision-targeted.

## References

[1] Rosenberg SA Restifo NP. Adoptive cell transfer as personalized immunotherapy for human cancer. Science. (2015) 348:62–8. doi: 10.1126/science.aaa4967, PMID: 25838374 PMC6295668

[2] Fefer A. Immunotherapy and chemotherapy of Moloney sarcoma virus-induced tumors in mice. Cancer Res. (1969) 29:2177–83. 5369675

[3] Fernandez-Cruz E Woda BA Feldman JD. Elimination of syngeneic sarcomas in rats by a subset of T lymphocytes. J Exp Med. (1980) 152:823–41. doi: 10.1084/jem.152.4.823, PMID: 6968337 PMC2185982

[4] Morgan DA Ruscetti FW Gallo R. Selective *in vitro* growth of T lymphocytes from normal human bone marrows. Science. (1976) 193:1007–8. doi: 10.1126/science.181845, PMID: 181845

[5] Kessels HW Wolkers MC van den Boom MD van der Valk MA Schumacher TN. Immunotherapy through TCR gene transfer. Nat Immunol. (2001) 2:957–61. doi: 10.1038/ni1001-957, PMID: 11577349

[6] Morgan RA Dudley ME Wunderlich JR Hughes MS Yang JC Sherry RM. Cancer regression in patients after transfer of genetically engineered lymphocytes. Science. (2006) 314:126–9. doi: 10.1126/science.1129003, PMID: 16946036 PMC2267026

[7] Kochenderfer JN Wilson WH Janik JE Dudley ME Stetler-Stevenson M Feldman SA. Eradication of B-lineage cells and regression of lymphoma in a patient treated with autologous T cells genetically engineered to recognize CD19. Blood. (2010) 116:4099–102. doi: 10.1182/blood-2010-04-281931, PMID: 20668228 PMC2993617

[8] Rosenberg SA Lotze MT Muul LM Leitman S Chang AE Ettinghausen SE. Observations on the systemic administration of autologous lymphokine-activated killer cells and recombinant interleukin-2 to patients with metastatic cancer. N Engl J Med. (1985) 313:1485–92. doi: 10.1056/NEJM198512053132327, PMID: 3903508

[9] Grimm EA Mazumder A Zhang HZ Rosenberg SA. Lymphokine-activated killer cell phenomenon. Lysis of natural killer-resistant fresh solid tumor cells by interleukin 2-activated autologous human peripheral blood lymphocytes. J Exp Med. (1982) 155:1823–41. doi: 10.1084/jem.155.6.1823, PMID: 6176669 PMC2186695

[10] Bauer S Groh V Wu J Steinle A Phillips JH Lanier LL. Activation of NK cells and T cells by NKG2D, a receptor for stress-inducible MICA. Science. (1999) 285:727–9. doi: 10.1126/science.285.5428.727, PMID: 10426993

[11] Chaoul N Lauricella E Giglio A D’Angelo G Ganini C Cives M. The future of cellular therapy for the treatment of renal cell carcinoma. Expert Opin Biol Ther. (2024) 2024:1–15. doi: 10.1080/14712598.2024.2418321, PMID: 39485013

[12] Topalian SL Solomon D Avis FP Chang AE Freerksen DL Linehan WM. Immunotherapy of patients with advanced cancer using tumor-infiltrating lymphocytes and recombinant interleukin-2: a pilot study. J Clin Oncol. (1988) 6:839–53. doi: 10.1200/JCO.1988.6.5.839, PMID: 3259261

[13] Kumar A Watkins R Vilgelm AE. Cell therapy with TILs: training and taming T cells to fight cancer. Front Immunol. (2021) 12:690499. doi: 10.3389/fimmu.2021.690499, PMID: 34140957 PMC8204054

[14] Seitter SJ Sherry RM Yang JC Robbins PF Shindorf ML Copeland AR. Impact of prior treatment on the efficacy of adoptive transfer of tumor-infiltrating lymphocytes in patients with metastatic melanoma. Clin Cancer Res. (2021) 27:5289–98. doi: 10.1158/1078-0432.CCR-21-1171, PMID: 34413159 PMC8857302

[15] Dafni U Michielin O Lluesma SM Tsourti Z Polydoropoulou V Karlis D. Efficacy of adoptive therapy with tumor-infiltrating lymphocytes and recombinant interleukin-2 in advanced cutaneous melanoma: a systematic review and meta-analysis. Ann Oncol. (2019) 30:1902–13. doi: 10.1093/annonc/mdz398, PMID: 31566658

[16] Parums DV. Editorial: first regulatory approval for adoptive cell therapy with autologous tumor-infiltrating lymphocytes (TILs) - lifileucel (Amtagvi). Med Sci Monit. (2024) 30:e944927. doi: 10.12659/MSM.944927, PMID: 38689550 PMC11071689

[17] Klobuch S Seijkens TTP Schumacher TN Haanen J. Tumour-infiltrating lymphocyte therapy for patients with advanced-stage melanoma. Nat Rev Clin Oncol. (2024) 21:173–84. doi: 10.1038/s41571-023-00848-w, PMID: 38191921

[18] Jazaeri AA Zsiros E Amaria RN Artz AS Edwards RP Wenham RM. Safety and efficacy of adoptive cell transfer using autologous tumor infiltrating lymphocytes (LN-145) for treatment of recurrent, metastatic, or persistent cervical carcinoma. J Clin Oncol. (2019) 37(15_suppl):2538-.

[19] Dudley ME Gross CA Somerville RP Hong Y Schaub NP Rosati SF. Randomized selection design trial evaluating CD8+-enriched versus unselected tumor-infiltrating lymphocytes for adoptive cell therapy for patients with melanoma. J Clin Oncol. (2013) 31:2152–9. doi: 10.1200/JCO.2012.46.6441, PMID: 23650429 PMC3731980

[20] Schmidt-Wolf IG Negrin RS Kiem HP Blume KG Weissman IL. Use of a SCID mouse/human lymphoma model to evaluate cytokine-induced killer cells with potent antitumor cell activity. J Exp Med. (1991) 174:139–49. doi: 10.1084/jem.174.1.139, PMID: 1711560 PMC2118875

[21] Yron I Wood TA Jr. Spiess PJ Rosenberg SA. *In vitro* growth of murine T cells. V. The isolation and growth of lymphoid cells infiltrating syngeneic solid tumors. J Immunol. (1980) 125:238–45. doi: 10.4049/jimmunol.125.1.238, PMID: 6966652

[22] Lotze MT Line BR Mathisen DJ Rosenberg SA. The *in vivo* distribution of autologous human and murine lymphoid cells grown in T cell growth factor (TCGF): implications for the adoptive immunotherapy of tumors. J Immunol. (1980) 125:1487–93. doi: 10.4049/jimmunol.125.4.1487, PMID: 6967906

[23] Jiang J Wu C Lu B. Cytokine-induced killer cells promote antitumor immunity. J Transl Med. (2013) 11:83. doi: 10.1186/1479-5876-11-83, PMID: 23536996 PMC3617047

[24] Ochoa AC Gromo G Alter BJ Sondel PM Bach FH. Long-term growth of lymphokine-activated killer (LAK) cells: role of anti-CD3, beta-IL 1, interferon-gamma and -beta. J Immunol. (1987) 138:2728–33. doi: 10.4049/jimmunol.138.8.2728, PMID: 2435804

[25] Introna M Borleri G Conti E Franceschetti M Barbui AM Broady R. Repeated infusions of donor-derived cytokine-induced killer cells in patients relapsing after allogeneic stem cell transplantation: a phase I study. Haematologica. (2007) 92:952–9. doi: 10.3324/haematol.11132, PMID: 17606446

[26] Leemhuis T Wells S Scheffold C Edinger M Negrin RS. A phase I trial of autologous cytokine-induced killer cells for the treatment of relapsed Hodgkin disease and non-Hodgkin lymphoma. Biol Blood Marrow Transplant. (2005) 11:181–7. doi: 10.1016/j.bbmt.2004.11.019, PMID: 15744236

[27] Laport GG Sheehan K Baker J Armstrong R Wong RM Lowsky R. Adoptive immunotherapy with cytokine-induced killer cells for patients with relapsed hematologic Malignancies after allogeneic hematopoietic cell transplantation. Biol Blood Marrow Transplant. (2011) 17:1679–87. doi: 10.1016/j.bbmt.2011.05.012, PMID: 21664472 PMC3175285

[28] Zhang Y Schmidt-Wolf IGH. Ten-year update of the international registry on cytokine-induced killer cells in cancer immunotherapy. J Cell Physiol. (2020) 235:9291–303. doi: 10.1002/jcp.29827, PMID: 32484595

[29] Jiang J Xu N Wu C Deng H Lu M Li M. Treatment of advanced gastric cancer by chemotherapy combined with autologous cytokine-induced killer cells. Anticancer Res. (2006) 26:2237–42., PMID: 16821594

[30] Wu C Jiang J Shi L Xu N. Prospective study of chemotherapy in combination with cytokine-induced killer cells in patients suffering from advanced non-small cell lung cancer. Anticancer Res. (. 2008) ;28:3997–4002., PMID: 19192663

[31] Huang ZM Li W Li S Gao F Zhou QM Wu FM. Cytokine-induced killer cells in combination with transcatheter arterial chemoembolization and radiofrequency ablation for hepatocellular carcinoma patients. J Immunother. (2013) 36:287–93. doi: 10.1097/CJI.0b013e3182948452, PMID: 23719239

[32] Takayama T Sekine T Makuuchi M Yamasaki S Kosuge T Yamamoto J. Adoptive immunotherapy to lower postsurgical recurrence rates of hepatocellular carcinoma: a randomised trial. Lancet. (2000) 356:802–7. doi: 10.1016/S0140-6736(00)02654-4, PMID: 11022927

[33] Zhou ZQ Zhao JJ Pan QZ Chen CL Liu Y Tang Y. PD-L1 expression is a predictive biomarker for CIK cell-based immunotherapy in postoperative patients with breast cancer. J Immunother Cancer. (2019) 7:228. doi: 10.1186/s40425-019-0696-8, PMID: 31455411 PMC6712838

[34] Li Y Pan K Liu LZ Li YQ Gu MF Zhang H. Sequential cytokine-induced killer cell immunotherapy enhances the efficacy of the gemcitabine plus cisplatin chemotherapy regimen for metastatic nasopharyngeal carcinoma. PloS One. (2015) 10:e0130620. doi: 10.1371/journal.pone.0130620, PMID: 26098948 PMC4476660

[35] Jiang W Wang Z Luo Q Dai Z Zhu J Tao X. Combined immunotherapy with dendritic cells and cytokine-induced killer cells for solid tumors: a systematic review and meta-analysis of randomized controlled trials. J Transl Med. (2024) 22:1122. doi: 10.1186/s12967-024-05940-y, PMID: 39707416 PMC11662567

[36] Wang Y Xiang Y Xin VW Wang XW Peng XC Liu XQ. Dendritic cell biology and its role in tumor immunotherapy. J Hematol Oncol. (2020) 13:107. doi: 10.1186/s13045-020-00939-6, PMID: 32746880 PMC7397618

[37] Rossjohn J Gras S Miles JJ Turner SJ Godfrey DI McCluskey J. T cell antigen receptor recognition of antigen-presenting molecules. Annu Rev Immunol. (2015) 33:169–200. doi: 10.1146/annurev-immunol-032414-112334, PMID: 25493333

[38] Tkach M Kowal J Zucchetti AE Enserink L Jouve M Lankar D. Qualitative differences in T-cell activation by dendritic cell-derived extracellular vesicle subtypes. EMBO J. (2017) 36:3012–28. doi: 10.15252/embj.201696003, PMID: 28923825 PMC5641679

[39] Pitt JM André F Amigorena S Soria JC Eggermont A Kroemer G. Dendritic cell-derived exosomes for cancer therapy. J Clin Invest. (2016) 126:1224–32. doi: 10.1172/JCI81137, PMID: 27035813 PMC4811123

[40] Liu Z Ravindranathan R Kalinski P Guo ZS Bartlett DL. Rational combination of oncolytic vaccinia virus and PD-L1 blockade works synergistically to enhance therapeutic efficacy. Nat Commun. (2017) 8:14754. doi: 10.1038/ncomms14754, PMID: 28345650 PMC5378974

[41] Rao Q Zuo B Lu Z Gao X You A Wu C. Tumor-derived exosomes elicit tumor suppression in murine hepatocellular carcinoma models and humans *in vitro* . Hepatology. (2016) 64:456–72. doi: 10.1002/hep.28549, PMID: 26990897

[42] Halim TY Hwang YY Scanlon ST Zaghouani H Garbi N Fallon PG. Group 2 innate lymphoid cells license dendritic cells to potentiate memory TH2 cell responses. Nat Immunol. (2016) 17:57–64. doi: 10.1038/ni.3294, PMID: 26523868 PMC4685755

[43] Banchereau J Steinman RM. Dendritic cells and the control of immunity. Nature. (1998) 392:245–52. doi: 10.1038/32588., PMID: 9521319

[44] Šamija I Fröbe A. CHALLENGES IN MANIPULATING IMMUNE SYSTEM TO TREAT PROSTATE CANCER. Acta Clin Croat. (2019) 58:76–81. doi: 10.20471/acc.2019.58.s2.13, PMID: 34975203 PMC8693557

[45] Wang W Zou C Liu X He L Cao Z Zhu M. Biomimetic dendritic cell-based nanovaccines for reprogramming the immune microenvironment to boost tumor immunotherapy. ACS Nano. (2024) 18(50):34063–76. doi: 10.1021/acsnano.4c09653, PMID: 39625243

[46] Bol KF Schreibelt G Bloemendal M van Willigen WW Hins-de Bree S de Goede AL. Adjuvant dendritic cell therapy in stage IIIB/C melanoma: the MIND-DC randomized phase III trial. Nat Commun. (2024) 15:1632. doi: 10.1038/s41467-024-45358-0, PMID: 38395969 PMC10891118

[47] Ascic E Åkerström F Sreekumar Nair M Rosa A Kurochkin I Zimmermannova O. *In vivo* dendritic cell reprogramming for cancer immunotherapy. Science. (2024) 386:eadn9083. doi: 10.1126/science.adn9083, PMID: 39236156 PMC7616765

[48] Dan J Cai J Zhong Y Wang C Huang S Zeng Y. Oncolytic virus M1 functions as a bifunctional checkpoint inhibitor to enhance the antitumor activity of DC vaccine. Cell Rep Med. (2023) 4:101229. doi: 10.1016/j.xcrm.2023.101229, PMID: 37820722 PMC10591054

[49] Zhao X Shao S Hu L. The recent advancement of TCR-T cell therapies for cancer treatment. Acta Biochim Biophys Sin (Shanghai). (2024) 56:663–74. doi: 10.3724/abbs.2024034, PMID: 38557898 PMC11187488

[50] Palacios EH Weiss A. Function of the Src-family kinases, Lck and Fyn, in T-cell development and activation. Oncogene. (2004) 23:7990–8000. doi: 10.1038/sj.onc.1208074, PMID: 15489916

[51] Jaeger AM Stopfer LE Ahn R Sanders EA Sandel DA Freed-Pastor WA. Deciphering the immunopeptidome *in vivo* reveals new tumour antigens. Nature. (2022) 607:149–55., PMID: 35705813 10.1038/s41586-022-04839-2 PMC9945857

[52] Neefjes J Jongsma ML Paul P Bakke O. Towards a systems understanding of MHC class I and MHC class II antigen presentation. Nat Rev Immunol. (2011) 11:823–36. doi: 10.1038/nri3084, PMID: 22076556

[53] Harris DT Kranz DM. Adoptive T cell therapies: A comparison of T cell receptors and chimeric antigen receptors. Trends Pharmacol Sci. (2016) 37:220–30. doi: 10.1016/j.tips.2015.11.004, PMID: 26705086 PMC4764454

[54] Baulu E Gardet C Chuvin N Depil S. TCR-engineered T cell therapy in solid tumors: State of the art and perspectives. Sci Adv. (2023) 9:eadf3700. doi: 10.1126/sciadv.adf3700, PMID: 36791198 PMC9931212

[55] Zhang J Wang L. The emerging world of TCR-T cell trials against cancer: A systematic review. Technol Cancer Res Treat. (2019) 18:1533033819831068. doi: 10.1177/1533033819831068, PMID: 30798772 PMC6391541

[56] Nagarsheth NB Norberg SM Sinkoe AL Adhikary S Meyer TJ Lack JB. TCR-engineered T cells targeting E7 for patients with metastatic HPV-associated epithelial cancers. Nat Med. (2021) 27:419–25. doi: 10.1038/s41591-020-01225-1, PMID: 33558725 PMC9620481

[57] Gaillard A Matet A Rodrigues M. New drug approval: Tebentafusp for treatment of metastatic uveal melanoma HLA A*02:01-positive patients. Bull Cancer. (2023) 110:9–10. doi: 10.1016/j.bulcan.2022.10.001, PMID: 36357199

[58] Keam SJ. Afamitresgene autoleucel: first approval. Mol Diagn Ther. (2024) 28:861–6. doi: 10.1007/s40291-024-00749-3, PMID: 39404764

[59] Pan Q Weng D Liu J Han Z Ou Y Xu B. Phase 1 clinical trial to assess safety and efficacy of NY-ESO-1-specific TCR T cells in HLA-A∗02:01 patients with advanced soft tissue sarcoma. Cell Rep Med. (2023) 4:101133. doi: 10.1016/j.xcrm.2023.101133, PMID: 37586317 PMC10439245

[60] Zhao Q Jiang Y Xiang S Kaboli PJ Shen J Zhao Y. Engineered TCR-T cell immunotherapy in anticancer precision medicine: pros and cons. Front Immunol. (2021) 12:658753. doi: 10.3389/fimmu.2021.658753, PMID: 33859650 PMC8042275

[61] Enblad G Karlsson H Loskog AS. CAR T-cell therapy: the role of physical barriers and immunosuppression in lymphoma. Hum Gene Ther. (2015) 26:498–505. doi: 10.1089/hum.2015.054, PMID: 26230974 PMC4554546

[62] Levine BL. Performance-enhancing drugs: design and production of redirected chimeric antigen receptor (CAR) T cells. Cancer Gene Ther. (2015) 22:79–84. doi: 10.1038/cgt.2015.5, PMID: 25675873

[63] Shirzadian M Moori S Rabbani R Rahbarizadeh F. SynNotch CAR-T cell, when synthetic biology and immunology meet again. Front Immunol. (2025) 16:1545270. doi: 10.3389/fimmu.2025.1545270, PMID: 40308611 PMC12040928

[64] Pievani A Biondi M Tettamanti S Biondi A Dotti G Serafini M. CARs are sharpening their weapons. J Immunother Cancer. (2024) 12(1):6. doi: 10.1136/jitc-2023-008275, PMID: 38296592 PMC10831441

[65] Turtle CJ Hanafi LA Berger C Gooley TA Cherian S Hudecek M. CD19 CAR-T cells of defined CD4+:CD8+ composition in adult B cell ALL patients. J Clin Invest. (2016) 126:2123–38. doi: 10.1172/JCI85309, PMID: 27111235 PMC4887159

[66] Lee DW Kochenderfer JN Stetler-Stevenson M Cui YK Delbrook C Feldman SA. T cells expressing CD19 chimeric antigen receptors for acute lymphoblastic leukaemia in children and young adults: a phase 1 dose-escalation trial. Lancet. (2015) 385:517–28. doi: 10.1016/S0140-6736(14)61403-3, PMID: 25319501 PMC7065359

[67] Zhang WY Wang Y Guo YL Dai HR Yang QM Zhang YJ. Treatment of CD20-directed Chimeric Antigen Receptor-modified T cells in patients with relapsed or refractory B-cell non-Hodgkin lymphoma: an early phase IIa trial report. Signal Transduct Target Ther. (2016) 1:16002. doi: 10.1038/sigtrans.2016.2, PMID: 29263894 PMC5661644

[68] Wang CM Wu ZQ Wang Y Guo YL Dai HR Wang XH. Autologous T cells expressing CD30 chimeric antigen receptors for relapsed or refractory hodgkin lymphoma: an open-label phase I trial. Clin Cancer Res. (2017) 23:1156–66. doi: 10.1158/1078-0432.CCR-16-1365, PMID: 27582488

[69] Maude SL Frey N Shaw PA Aplenc R Barrett DM Bunin NJ. Chimeric antigen receptor T cells for sustained remissions in leukemia. N Engl J Med. (2014) 371:1507–17. doi: 10.1056/NEJMoa1407222, PMID: 25317870 PMC4267531

[70] Morabito F Martino EA Nizzoli ME Talami A Pozzi S Martino M. Comparative analysis of bispecific antibodies and CAR T-cell therapy in follicular lymphoma. Eur J Haematol. (2025) 114:4–16. doi: 10.1111/ejh.14335, PMID: 39462177 PMC11613673

[71] Brijs J Van Ham J Dubois B Sinap F Vergote V Dierickx D. Single center, real-world retrospective study of CAR-T cell therapy for relapsed/refractory large B-cell lymphoma beyond second line: five-year results at the University Hospitals Leuven. Acta Clin Belg. (2024) 79:276–84. doi: 10.1080/17843286.2024.2399365, PMID: 39415456

[72] Ying Z Yang H Guo Y Li W Zou D Zhou D. Relmacabtagene autoleucel (relma-cel) CD19 CAR-T therapy for adults with heavily pretreated relapsed/refractory large B-cell lymphoma in China. Cancer Med. (2021) 10:999–1011. doi: 10.1002/cam4.3686, PMID: 33382529 PMC7897944

[73] Kochenderfer JN Dudley ME Kassim SH Somerville RP Carpenter RO Stetler-Stevenson M. Chemotherapy-refractory diffuse large B-cell lymphoma and indolent B-cell Malignancies can be effectively treated with autologous T cells expressing an anti-CD19 chimeric antigen receptor. J Clin Oncol. (2015) 33:540–9. doi: 10.1200/JCO.2014.56.2025, PMID: 25154820 PMC4322257

[74] Zhao Z Chen Y Francisco NM Zhang Y Wu M. The application of CAR-T cell therapy in hematological Malignancies: advantages and challenges. Acta Pharm Sin B. (2018) 8:539–51. doi: 10.1016/j.apsb.2018.03.001, PMID: 30109179 PMC6090008

[75] Kakarla S Gottschalk S. CAR T cells for solid tumors: armed and ready to go? Cancer J. (2014) 20:151–5. doi: 10.1097/PPO.0000000000000032, PMID: 24667962 PMC4050065

[76] Hillerdal V Essand M. Chimeric antigen receptor-engineered T cells for the treatment of metastatic prostate cancer. BioDrugs. (2015) 29:75–89. doi: 10.1007/s40259-015-0122-9, PMID: 25859858 PMC4544486

[77] Qi C Liu C Gong J Liu D Wang X Zhang P. Claudin18.2-specific CAR T cells in gastrointestinal cancers: phase 1 trial final results. Nat Med. (2024) 30:2224–34. doi: 10.1038/s41591-024-03037-z, PMID: 38830992

[78] Shen Y Ma C Li X Li X Wu Y Yang T. Generation of B7-H3 isoform regulated by ANXA2/NSUN2/YBX1 axis in human glioma. J Cell Mol Med. (2024) 28:e18575. doi: 10.1111/jcmm.18575, PMID: 39048916 PMC11269050

[79] Ramalingam PS Premkumar T Sundararajan V Hussain MS Arumugam S. Design and development of dual targeting CAR protein for the development of CAR T-cell therapy against KRAS mutated pancreatic ductal adenocarcinoma using computational approaches. Discov Oncol. (2024) 15:592. doi: 10.1007/s12672-024-01455-6, PMID: 39453574 PMC11511808

[80] Jilani S Saco JD Mugarza E Pujol-Morcillo A Chokry J Ng C. CAR-T cell therapy targeting surface expression of TYRP1 to treat cutaneous and rare melanoma subtypes. Nat Commun. (2024) 15:1244. doi: 10.1038/s41467-024-45221-2, PMID: 38336975 PMC10858182

[81] Hong G Chen X Sun X Zhou M Liu B Li Z. Effect of autologous NK cell immunotherapy on advanced lung adenocarcinoma with EGFR mutations. Precis Clin Med. (2019) 2:235–45. doi: 10.1093/pcmedi/pbz023, PMID: 35693880 PMC8985770

[82] Valipour B Velaei K Abedelahi A Karimipour M Darabi M Charoudeh HN. NK cells: An attractive candidate for cancer therapy. J Cell Physiol. (2019) 234:19352–65. doi: 10.1002/jcp.28657, PMID: 30993712

[83] Mikhailova VA Sokolov DI Grebenkina PV Bazhenov DO Nikolaenkov IP Kogan IY. Apoptotic receptors and CD107a expression by NK cells in an interaction model with trophoblast cells. Curr Issues Mol Biol. (2024) 46:8945–57. doi: 10.3390/cimb46080528, PMID: 39194745 PMC11352607

[84] Fares J Davis ZB Rechberger JS Toll SA Schwartz JD Daniels DJ. Advances in NK cell therapy for brain tumors. NPJ Precis Oncol. (2023) 7:17. doi: 10.1038/s41698-023-00356-1, PMID: 36792722 PMC9932101

[85] Peng XR Cheng J. Research progress of chimeric antigen receptor modified NK cells in the treatment of acute myeloid leukemia –review. Zhongguo Shi Yan Xue Ye Xue Za Zhi. (2023) 31:1905–9. doi: 10.19746/j.cnki.issn.1009-2137.2023.06.048, PMID: 38071081

[86] Kumar A Emdad L Das SK Fisher PB. Recent advances and progress in immunotherapy of solid cancers. Adv Cancer Res. (2024) 164:111–90. doi: 10.1016/bs.acr.2024.05.004, PMID: 39306365

[87] Laskowski TJ Biederstädt A Rezvani K. Natural killer cells in antitumour adoptive cell immunotherapy. Nat Rev Cancer. (2022) 22:557–75. doi: 10.1038/s41568-022-00491-0, PMID: 35879429 PMC9309992

[88] Park H Kim G Kim N Ha S Yim H. Efficacy and safety of natural killer cell therapy in patients with solid tumors: a systematic review and meta-analysis. Front Immunol. (2024) 15:1454427. doi: 10.3389/fimmu.2024.1454427, PMID: 39478866 PMC11522797

[89] Maalej KM Merhi M Inchakalody VP Mestiri S Alam M Maccalli C. CAR-cell therapy in the era of solid tumor treatment: current challenges and emerging therapeutic advances. Mol Cancer. (2023) 22:20. doi: 10.1186/s12943-023-01723-z, PMID: 36717905 PMC9885707

[90] Xie G Dong H Liang Y Ham JD Rizwan R Chen J. CAR-NK cells: A promising cellular immunotherapy for cancer. EBioMedicine. (2020) 59:102975. doi: 10.1016/j.ebiom.2020.102975, PMID: 32853984 PMC7452675

[91] Myers JA Miller JS. Exploring the NK cell platform for cancer immunotherapy. Nat Rev Clin Oncol. (2021) 18:85–100. doi: 10.1038/s41571-020-0426-7, PMID: 32934330 PMC8316981

[92] Ahmadvand M Barough MS Barkhordar M Faridfar A Ghaderi A Jalaeikhoo H. Phase I non-randomized clinical trial of allogeneic natural killer cells infusion in acute myeloid leukemia patients. BMC Cancer. (2023) 23:1090. doi: 10.1186/s12885-023-11610-x, PMID: 37950209 PMC10636850

[93] Pu J Liu T Sharma A Jiang L Wei F Ren X. Advances in adoptive cellular immunotherapy and therapeutic breakthroughs in multiple myeloma. Exp Hematol Oncol. (2024) 13:105. doi: 10.1186/s40164-024-00576-6, PMID: 39468695 PMC11514856

[94] Bae WK Lee BC Kim HJ Lee JJ Chung IJ Cho SB. A phase I study of locoregional high-dose autologous natural killer cell therapy with hepatic arterial infusion chemotherapy in patients with locally advanced hepatocellular carcinoma. Front Immunol. (2022) 13:879452. doi: 10.3389/fimmu.2022.879452, PMID: 35720374 PMC9202498

[95] Jensen SS Hartling HJ Tingstedt JL Larsen TK Nielsen SD Pedersen C. HIV-specific ADCC improves after antiretroviral therapy and correlates with normalization of the NK cell phenotype. J Acquir Immune Defic Syndr. (2015) 68:103–11. doi: 10.1097/QAI.0000000000000429, PMID: 25394194

[96] Sun B da Costa KAS Alrubayyi A Kokici J Fisher-Pearson N Hussain N. HIV/HBV coinfection remodels the immune landscape and natural killer cell ADCC functional responses. Hepatology. (2024) 80:649–63. doi: 10.1097/HEP.0000000000000877, PMID: 38687604 PMC11782918

[97] Ghasemzadeh M Ghasemzadeh A Hosseini E. Exhausted NK cells and cytokine storms in COVID-19: Whether NK cell therapy could be a therapeutic choice. Hum Immunol. (2022) 83:86–98. doi: 10.1016/j.humimm.2021.09.004, PMID: 34583856 PMC8423992

[98] Luo J Guo M Huang M Liu Y Qian Y Liu Q. Neoleukin-2/15-armored CAR-NK cells sustain superior therapeutic efficacy in solid tumors via c-Myc/NRF1 activation. Signal Transduct Target Ther. (2025) 10:78. doi: 10.1038/s41392-025-02158-2, PMID: 40025022 PMC11873268

[99] Hermanson DL Bendzick L Pribyl L McCullar V Vogel RI Miller JS. Induced pluripotent stem cell-derived natural killer cells for treatment of ovarian cancer. Stem Cells. (2016) 34:93–101. doi: 10.1002/stem.2230, PMID: 26503833 PMC4713309

[100] Qiao W Dong P Chen H Zhang J. Advances in induced pluripotent stem cell-derived natural killer cell therapy. Cells. (2024) 13(23):1976. doi: 10.3390/cells13231976, PMID: 39682724 PMC11640743

[101] Rea A Santana-Hernández S Villanueva J Sanvicente-García M Cabo M Suarez-Olmos J. Enhancing human NK cell antitumor function by knocking out SMAD4 to counteract TGFβ and activin A suppression. Nat Immunol. (2025) 26(4):582–94., PMID: 40119192 10.1038/s41590-025-02103-z PMC11957989

[102] Pan K Farrukh H Chittepu V Xu H Pan CX Zhu Z. CAR race to cancer immunotherapy: from CAR T, CAR NK to CAR macrophage therapy. J Exp Clin Cancer Res. (2022) 41:119. doi: 10.1186/s13046-022-02327-z, PMID: 35361234 PMC8969382

[103] Chang Y Jin G Luo W Luo Q Jung J Hummel SN. Engineered human pluripotent stem cell-derived natural killer cells with PD-L1 responsive immunological memory for enhanced immunotherapeutic efficacy. Bioact Mater. (2023) 27:168–80. doi: 10.1016/j.bioactmat.2023.03.018, PMID: 37091063 PMC10113709

[104] Yadav S Priya A Borade DR Agrawal-Rajput R. Macrophage subsets and their role: co-relation with colony-stimulating factor-1 receptor and clinical relevance. Immunol Res. (2023) 71:130–52. doi: 10.1007/s12026-022-09330-8, PMID: 36266603 PMC9589538

[105] Cheng XL Ding F Li H Tan XQ Liu X Cao JM. Activation of AMPA receptor promotes TNF-α release via the ROS-cSrc-NFκB signaling cascade in RAW264.7 macrophages. Biochem Biophys Res Commun. (2015) 461:275–80. doi: 10.1016/j.bbrc.2015.04.015, PMID: 25871799

[106] Nahrendorf M Swirski FK. Abandoning M1/M2 for a network model of macrophage function. Circ Res. (2016) 119:414–7. doi: 10.1161/CIRCRESAHA.116.309194, PMID: 27458196 PMC4965179

[107] Fontana F Iacoponi F Orlando F Pratellesi T Cafarelli A Ricotti L. Low-intensity pulsed ultrasound increases neurotrophic factors secretion and suppresses inflammation in*in vitro*models of peripheral neuropathies. J Neural Eng. (2023) 20(2):1741–2552. doi: 10.1088/1741-2552/acc54e, PMID: 36930982

[108] Huo Y Zhang H Sa L Zheng W He Y Lyu H. M1 polarization enhances the antitumor activity of chimeric antigen receptor macrophages in solid tumors. J Transl Med. (2023) 21:225. doi: 10.1186/s12967-023-04061-2, PMID: 36978075 PMC10044396

[109] Klichinsky M Ruella M Shestova O Lu XM Best A Zeeman M. Human chimeric antigen receptor macrophages for cancer immunotherapy. Nat Biotechnol. (2020) 38:947–53. doi: 10.1038/s41587-020-0462-y, PMID: 32361713 PMC7883632

[110] Wang S Yang Y Ma P Zha Y Zhang J Lei A. CAR-macrophage: An extensive immune enhancer to fight cancer. EBioMedicine. (2022) 76:103873. doi: 10.1016/j.ebiom.2022.103873, PMID: 35152151 PMC8844597

[111] Lei A Yu H Lu S Lu H Ding X Tan T. A second-generation M1-polarized CAR macrophage with antitumor efficacy. Nat Immunol. (2024) 25:102–16. doi: 10.1038/s41590-023-01687-8, PMID: 38012418

[112] Kang M Lee SH Kwon M Byun J Kim D Kim C. Nanocomplex-mediated *in vivo* programming to chimeric antigen receptor-M1 macrophages for cancer therapy. Adv Mater. (2021) 33:e2103258. doi: 10.1002/adma.202103258, PMID: 34510559

[113] Zheng H Yang X Huang N Yuan S Li J Liu X. Chimeric antigen receptor macrophages targeting c-MET(CAR-M-c-MET) inhibit pancreatic cancer progression and improve cytotoxic chemotherapeutic efficacy. Mol Cancer. (2024) 23:270. doi: 10.1186/s12943-024-02184-8, PMID: 39643883 PMC11622543

[114] Reiss KA Angelos MG Dees EC Yuan Y Ueno NT Pohlmann PR. CAR-macrophage therapy for HER2-overexpressing advanced solid tumors: a phase 1 trial. Nat Med. (2025) 31:1171–82. doi: 10.1038/s41591-025-03495-z, PMID: 39920391

[115] Zhang L Tian L Dai X Yu H Wang J Lei A. Pluripotent stem cell-derived CAR-macrophage cells with antigen-dependent anti-cancer cell functions. J Hematol Oncol. (2020) 13:153. doi: 10.1186/s13045-020-00983-2, PMID: 33176869 PMC7656711

[116] Majzner RG Mackall CL. Clinical lessons learned from the first leg of the CAR T cell journey. Nat Med. (2019) 25:1341–55. doi: 10.1038/s41591-019-0564-6, PMID: 31501612

[117] Rezvani K Rouce R Liu E Shpall E. Engineering natural killer cells for cancer immunotherapy. Mol Ther. (2017) 25:1769–81. doi: 10.1016/j.ymthe.2017.06.012, PMID: 28668320 PMC5542803

[118] Andreesen R Hennemann B Krause SW. Adoptive immunotherapy of cancer using monocyte-derived macrophages: rationale, current status, and perspectives. J Leukoc Biol. (1998) 64:419–26. doi: 10.1002/jlb.64.4.419, PMID: 9766621

[119] Lu J Ma Y Li Q Xu Y Xue Y Xu S. CAR Macrophages: a promising novel immunotherapy for solid tumors and beyond. biomark Res. (2024) 12:86. doi: 10.1186/s40364-024-00637-2, PMID: 39175095 PMC11342599

[120] Chávez-Galán L Olleros ML Vesin D Garcia I. Much More than M1 and M2 Macrophages, There are also CD169(+) and TCR(+) Macrophages. Front Immunol. (2015) 6:263. doi: 10.3389/fimmu.2015.00263, PMID: 26074923 PMC4443739

[121] Chettri D Satapathy BP Yadav R Uttam V Jain A Prakash H. CAR-macrophages: tailoring cancer immunotherapy. Front Immunol. (2024) 15:1532833. doi: 10.3389/fimmu.2024.1532833, PMID: 39877364 PMC11772431

[122] Liu M Liu J Liang Z Dai K Gan J Wang Q. CAR-macrophages and CAR-T cells synergistically kill tumor cells *in vitro* . Cells. (2022) 11(22):3692. doi: 10.3390/cells11223692, PMID: 36429120 PMC9688246

[123] Tang Z Davidson D Li R Zhong MC Qian J Chen J. Inflammatory macrophages exploit unconventional pro-phagocytic integrins for phagocytosis and anti-tumor immunity. Cell Rep. (2021) 37:110111. doi: 10.1016/j.celrep.2021.110111, PMID: 34910922

[124] Zhou X Zhu D Wu D Li G Liang H Zhang W. Microneedle delivery of CAR-M-like engineered macrophages alleviates intervertebral disc degeneration through enhanced efferocytosis capacity. Cell Rep Med. (2025) 6:102079. doi: 10.1016/j.xcrm.2025.102079, PMID: 40199328 PMC12047514

[125] Ayala Ceja M Khericha M Harris CM Puig-Saus C Chen YY. CAR-T cell manufacturing: Major process parameters and next-generation strategies. J Exp Med. (2024) 221(2):e20230903. doi: 10.1084/jem.20230903, PMID: 38226974 PMC10791545

[126] Zhang J Jia Z Pan H Ma W Liu Y Tian X. From induced pluripotent stem cell (iPSC) to universal immune cells: literature review of advances in a new generation of tumor therapies. Transl Cancer Res. (2025) 14:2495–507. doi: 10.21037/tcr-24-1087, PMID: 40386273 PMC12079212

[127] Zhou L Li Y Zheng D Zheng Y Cui Y Qin L. Bispecific CAR-T cells targeting FAP and GPC3 have the potential to treat hepatocellular carcinoma. Mol Ther Oncol. (2024) 32:200817. doi: 10.1016/j.omton.2024.200817, PMID: 38882528 PMC11179089

[128] Simon S Bugos G Salter AI Riddell SR. Synthetic receptors for logic gated T cell recognition and function. Curr Opin Immunol. (2022) 74:9–17. doi: 10.1016/j.coi.2021.09.003, PMID: 34571290 PMC8901444

[129] Zhu B Yin H Zhang D Zhang M Chao X Scimeca L. Synthetic biology approaches for improving the specificity and efficacy of cancer immunotherapy. Cell Mol Immunol. (2024) 21:436–47. doi: 10.1038/s41423-024-01153-x, PMID: 38605087 PMC11061174

[130] Cai Y Gong M Zeng M Leng F Lv D Guo J. Immunopeptidomics-guided discovery and characterization of neoantigens for personalized cancer immunotherapy. Sci Adv. (2025) 11:eadv6445. doi: 10.1126/sciadv.adv6445, PMID: 40397742 PMC12094241

[131] Zhao X Wei L Zhang X. Computational methods and data resources for predicting tumor neoantigens. Brief Bioinform. (2025) 26(4):bbaf302. doi: 10.1093/bib/bbaf302, PMID: 40605273 PMC12222050

[132] Shahzadi M Rafique H Waheed A Naz H Waheed A Zokirova FR. Artificial intelligence for chimeric antigen receptor-based therapies: a comprehensive review of current applications and future perspectives. Ther Adv Vaccines Immunother. (2024) 12:25151355241305856. doi: 10.1177/25151355241305856, PMID: 39691280 PMC11650588

[133] Savanur MA Weinstein-Marom H Gross G. Implementing logic gates for safer immunotherapy of cancer. Front Immunol. (2021) 12 021. doi: 10.3389/fimmu.2021.780399, PMID: 34804073 PMC8600566

